# Malnutrition prevalence according to GLIM and its feasibility in geriatric patients: a prospective cross-sectional study

**DOI:** 10.1007/s00394-023-03323-5

**Published:** 2024-01-19

**Authors:** Maria Enge, Frida Ostonen Peelen, Rikke Lundsgaard Nielsen, Anne Marie Beck, Ann Ödlund Olin, Tommy Cederholm, Anne-Marie Boström, Ingvild Paur

**Affiliations:** 1 Department of Geriatric Medicine, Jakobsbergsgeriatriken, Stockholm, Sweden; 2 Theme Inflammation and Aging, Nursing Unit Aging, Karolinska University Hospital, Huddinge, Sweden; 3 Department of Clinical Research, Copenhagen University Hospital Amager and Hvidovre, Hvidovre, Denmark; 4 Dietetic and Nutritional Research Unit EATEN, Herlev and Gentofte University Hospital, Herlev, Denmark; 5 Department of Quality and Patient Safety, Karolinska University Hospital Stockholm, Stockholm, Sweden; 6 Department of Clinical Science, Intervention and Technology, Karolinska Institute, Stockholm, Sweden; 7 Theme Inflammation and Aging, Medical Unit Aging, Karolinska University Hospital, Huddinge, Sweden; 8 Clinical Nutrition and Metabolism, Department of Public Health and Caring Sciences, Uppsala University, Uppsala, Sweden; 9 Division of Nursing, Department of Neurobiology, Care Sciences and Society, Karolinska Institute, Stockholm, Sweden; 10 Stockholms Sjukhem, Research and Development Unit, Stockholm, Sweden; 11 Norwegian Advisory Unit On Disease-Related Undernutrition, Oslo, Norway; 12 Department of Clinical Service, Division of Cancer Medicine, Oslo University Hospital, Nydalen, Postbox 4950, 0424 Oslo, Norway; 13 Institute for Clinical Medicine, Clinical Nutrition Research Group, UiT the Arctic University of Norway, Tromsø, Norway

**Keywords:** Malnutrition, Geriatric patients, Hospital, Body composition, Feasibility, Global leadership initiative on malnutrition

## Abstract

**Purpose:**

In 2019, the Global Leadership Initiative on Malnutrition (GLIM) suggested a 2-step diagnostic format for malnutrition including screening and diagnosis. Prospective validation and feasibility studies, using the complete set of the five GLIM criteria, are needed. The aims of this study were to determine the prevalence of malnutrition, and investigate how the prevalence varied with mode of screening. Furthermore, we assessed the feasibility of GLIM in geriatric patients.

**Methods:**

Consecutive patients from two acute geriatric wards were included. For screening risk of malnutrition, the Mini Nutritional Assessment-Short Form (MNA-SF) or Malnutrition Screening Tool (MST) were used. In accordance with GLIM, a combination of phenotypic and etiologic criteria were required for the diagnosis of malnutrition. Feasibility was determined based on % data completeness, and above 80% completeness was considered feasible.

**Results:**

One hundred patients (mean age 82 years, 58% women) were included. After screening with MNA-SF malnutrition was confirmed by GLIM in 51%, as compared with 35% after screening with MST (*p* = 0.039). Corresponding prevalence was 58% with no prior screening. Using hand grip strength as a supportive measure for reduced muscle mass, 69% of the patients were malnourished. Feasibility varied between 70 and 100% for the different GLIM criteria, with calf circumference as a proxy for reduced muscle mass having the lowest feasibility.

**Conclusion:**

In acute geriatric patients, the prevalence of malnutrition according to GLIM varied depending on the screening tool used. In this setting, GLIM appears feasible, besides for the criterion of reduced muscle mass.

## Introduction

In older adults, malnutrition is frequent and is related to many negative consequences such as increased morbidity, mortality, physical and cognitive decline, increased length of hospital stay, higher hospital readmission rates and increased healthcare costs [1]. Due to the serious burden of malnutrition in older adults, both for the individual and for the health care system, is it of particular interest to detect and treat malnutrition in geriatric clinical practice [1].

In 2019 a consensus report from the global clinical nutrition community proposed the Global Leadership Initiative on Malnutrition (GLIM) criteria for the diagnosis of malnutrition [2]. Prior to GLIM, the use of different criteria to detect malnutrition resulted in large variations and uncertainties of the prevalence of malnutrition [2]. The initiative recommends a two-step approach for the malnutrition diagnosis: (1) nutritional screening to identify patients at risk of malnutrition using any validated screening tool and (2) nutritional assessment for the diagnosis and grading of malnutrition severity [2]. There is however no international consensus on a single best screening tool to be used in geriatric clinical practice. Previous studies with GLIM have used different screening tools, and some have used no screening tool prior to applying the GLIM criteria, and accordingly malnutrition rates vary to a great extent [3, 4]. The Mini Nutritional Assessment-short form (MNA-SF) has so far often been used to both screen and determine malnutrition in older adults [5].

Furthermore in GLIM, a combination of phenotypic and etiological criteria are required for the diagnosis of malnutrition [2]. The GLIM criteria has three phenotypic criteria (non-volitional weight loss, low body mass index, and reduced muscle mass) and two etiologic criteria (reduced food intake or assimilation, and inflammation or disease burden). To diagnose malnutrition at least one phenotypic criterion and one etiologic criterion must be present and all criteria should be assessed [2]. To our knowledge, using the complete set of the five GLIM criteria in clinical practice is not fully implemented in older patients. Thus, its feasibility in geriatric clinical practice is uncertain. However, the application of GLIM in clinical practice is evolving. Cederholm et al*.*, report in a literature review from august 2021 to august 2022, 40 publications that have applied the GLIM criteria in older patients [3]. Still the majority were retrospective, many did not apply all five GLIM criteria or used adaptation to the criteria [3] for example the EFFORT trial used hand grip strength as a proxy for muscle mass [6]. The GLIM consortium has suggested a prioritized flow chart for measuring muscle mass or body composition (BC) within GLIM [7]. Quantification or estimation of muscle mass using computerized tomography (CT), dual-energy x-ray absorptiometry (DXA), or bioelectrical impedance analysis (BIA) are the preferred methods, and anthropometric measures such as calf circumference (CC) or mid-upper arm circumference (MAC) are possible substitutes when neither CT, DXA or BIA are available [7]. However, none of these measurements of muscle mass are commonly integrated or used in geriatric clinical practice, partly due to lack of clinical experience and lack of equipment and evidence regarding age-adjusted cut-off values for muscle mass. Thus, it remains unknown whether the phenotypic criteria of reduced muscle mass is feasible in geriatric clinical practice.

Even though the GLIM criteria were launched in 2019, information is still needed on both the feasibility of using the full set GLIM criteria in prospective studies, and the actual prevalence of malnutrition in geriatric clinical practice. Therefore, the aims of this study were to: (1) to determine the prevalence of malnutrition using the complete set of GLIM criteria in geriatric patients, and investigate how the prevalence varied with mode of screening; (2) to assess the feasibility of GLIM for the diagnosis of malnutrition in geriatric patients.

## Methods

### Study design and setting

This prospective cross-sectional study included patients from two geriatric clinics in Stockholm in Sweden, and was registered at the Swedish Ethical Review Authority (Dnr 2022-01822-01). Both clinics handle acute geriatric admissions. This study follows the reporting guideline for cross-sectional studies: Strengthening the Reporting of Observational Studies in Epidemiology (STROBE) [8].

### Participants and recruitment

The patients in this study were recruited from one ward at each clinic consecutively over an eight week period. The researchers (M.E and F.O.P) invited participants who fulfilled both the inclusion and the exclusion criteria. The participants received written and verbal study information. To be eligible participants had to: agree to participate, be able to consent (for example speaking Swedish, no severe cognitive impairment), have an expected admission > 24 h, have no prior readmissions during the study period, have a life expectancy > 3 months and be ≥ 65 years.

### Data collection

Data collection was performed by the researchers within 1–3 days after admission, with the exception of the physical function measures which were performed within a week after admission. Variables were extracted from the electronic patient records (EPR) after inclusion or retrospectively. Measures of physical function and muscle mass were collected by trained healthcare personnel associated with the wards. Adverse events were registered.

### Screening for the risk of malnutrition

The validated screening tools Mini Nutritional Assessment short form (MNA-SF) and Malnutrition Screening Tool (MST) were used to identify participants at risk of malnutrition.

#### Mini nutritional assessment short form (MNA-SF)

MNA-SF is a well validated screening tool for older adults in hospitals [9]. It consists of six questions regarding food intake, weight loss, mobility, psychological stress/acute disease, neuropsychological problems and BMI and scores range from 0 to 14 points [10]. Nutritional status was rated as: normal (12–14 points), risk of malnutrition (8–11 points) and malnutrition (0–7 points). Due to the acute hospitalization, all patients were given the score 0 for the item psychological stress/acute disease.

#### Malnutrition screening tool (MST)

MST is a simple two-question screening tool for malnutrition validated in hospitalized older patients [11, 12]. It covers weight loss (score 0–4, increasing with the quantity of weight lost) and reduced food intake [score 0 (yes) − 1 (no)]. Malnutrition status was rated as: “not at risk of malnutrition”; score = 0–1, and; “at risk of malnutrition”; score = 2–5.

### Malnutrition diagnosis

The GLIM criteria were used for the diagnosis of malnutrition, with or without prior screening. The diagnosis of malnutrition with GLIM was defined as those participants fulfilling any combination of at least one phenotypic criterion and at least one etiologic criterion. All GLIM criteria were evaluated.

The phenotypic and etiological criteria were defined as follows:

#### For the phenotypic criterion, the following were considered

1) *Non-volitional weight loss*: Information on non-volitional weight loss was extracted from EPR and defined as a weight loss of > 5% within 6 months or a weight loss of > 10% within 2 years.

2) *Low BMI*: BMI was calculated with weight and height from EPR using the following equation: weight (kg)/[height (m) × height (m)]. BMI was defined as low if < 20 for those < 70 years, and < 22 for those > 70 years;

3) *Reduced muscle mass*: CC was measured, as recommended by GLIM, in cm by a tape at the widest part of the calf [7]. Care was taken not to compress the subcutaneous tissue when placing the measuring tape around the calf. The participants sat or lay with their knees bent at 90°. Measurements were performed for each leg and the highest value was registered. CC and applied cut-offs of < 33 cm for men and < 32 cm for women was based on results from a previous study [13]. If overweight or obesity was present, the measured value was reduced by 3 cm if BMI was 25–30 and 7 cm if BMI was above 30 [13]. If a patient had oedema (based on clinical observation) in lower extremities, MAC was measured using a tape at the midpoint of the upper arm instead. MAC less than 21 cm was defined as low muscle mass for both sexes [14].

#### Handgrip strength and 30 s chair stand test

Hand grip strength (HGS) and 30 s Chair Stand Test (30-s-chair stand) were investigated as supportive measures for muscle function, as recommended by GLIM [7]. HGS was measured by using a handgrip dynamometer. Baseline hydraulic hand dynamometer (Sample A) and Saehan hydraulic hand dynamometer (Sample B) were used as described in the manufacturer’s protocols. The HGS was measured with one punch and repeated three times on both hands. Maximum force trial with the dominant hand, and the highest value was recorded [15]. HGS was compared to cut-off values adapted for gender (females < 16 kg and males < 27 kg) [16].

For 30-s-chair stand, a chair with a straight back and solid seat at the height of 45 cm was used. The patient was instructed to sit on the chair with arms folded across their chest. For the test, the participant stood up and sat down as quickly and frequently as possible within 30 s, keeping both arms folded across the chest. The arms could be used for assistance or for safety if needed. The mode of chair stand (use of arms or not) and the number of stands during this period was counted [17]. The 30-s-chair stand was compared to cut-off values adapted for gender and age [17]. Normal range for the 30 s chair stand test were: women 65–69 years: 11–16; 70–79 years: 10–15; 80–84 years: 9–14; 85–89 years: 8–13; 90–94 years: 4–11; men 65–69 years: 12–18; 70–74 years: 12–17; 75–79 years: 11–17; 80–84 years: 10–15; 85–89 years: 8–14; 90–94 years: 7–12.

#### For the etiologic criterion, the following were considered:

(1)* Reduced food intake or assimilation:* Reduced food intake was assessed by average energy intake from three-day dietary records and defined as < 50% of calculated energy need. According to standard hospital procedure, the ward nurses performed three-day food records on the first three days of admission. The standardized dietary record is designed to estimate caloric intake based on information from the procured meal supplier and the Swedish Food Agency’s food database (https://soknaringsinnehall.livsmedelsverket.se/). Energy intake from enteral and parenteral nutrition was added if provided. Energy requirements were calculated on weight, height, and activity level (mobility). Based on the mobility question in MNA-SF, mobility was registered as either bed-ridden or able to move around at the ward. Energy need was calculated from 25 kcal/ kg if bed-ridden and 30 kcal/ kg if able to move around. Adjusted calculations were made if BMI was above 25 (weight in BMI 25 + 25% of overshooting weight*25 or 30 kcal) [18]. Data about food intake were limited to three days, so reduced food intake was defined as “ < 50% of energy requirements during three days “.

Food assimilation was evaluated on information from EPR about dysphagia, nausea/vomiting, diarrhea and constipation.

(2) *Disease burden/inflammation*: To evaluate disease burden/inflammation, clinical diagnosis of acute and chronic inflammatory diseases were extracted from EPR and included; major infections, burns, trauma (example hip fracture) or closed head injury and other acute disease-/injury -related conditions associated with mild to moderate inflammation. Chronic disease-related mild to moderate inflammation (malignant disease, chronic obstructive pulmonary disease, congestive heart failure, chronic renal disease or any disease with chronic or recurrent inflammation). CRP was used as a supportive measure in cases of uncertainty. Presence of inflammation was defined as Plasma C-reactive protein (CRP) > 10 mg/L [19]. CRP was measured in standard clinical hospital laboratory practice.

#### Severity of malnutrition

Lastly, the severity of malnutrition was determined. Stage 1 (moderate malnutrition) required at least one phenotypic criterion: (1) 5%-10% weight loss within the past 6 months or 10%-20% from 6 months to 2 years; (2) low BMI corresponding to < 20 kg/m2 if < 70 years old, or < 22 kg/m2 if > 70 years old; (3) reduced muscle mass defined as CC < 33 cm for men and < 32 cm for women or mid-arm circumference < 21 cm. Stage 2 (severe malnutrition) required at least one phenotypic criterion: (1) > 10% weight loss within the past 6 months or > 20% beyond 6 months; (2) low BMI corresponding to < 18.5 kg/m^2^ if < 70 years or < 20 kg/m2 if > 70 years.

### Feasibility

The completeness of the datasets was used as a measure of feasibility. In addition, adverse events were documented. Data completeness of ≥ 80% was required for an outcome to be considered for a definitive trial [20].

### Sample size and statistics

To detect the difference in proportion of malnourished patients expected to be 64% in the current project (based on two other populations of geriatric patients) and with a significance level of 5% and power of 80%, a sample size of 98 is needed [21, 22].

Data was handled and analyzed using SPSS and Microsoft Excel. Descriptive statistics were used to describe baseline characteristics and malnutrition prevalence according to GLIM. When normally distributed continuous variables are presented as means with standard deviations (SD) and as median and interquartile range when not normally distributed. Chi-squared test, Fisher-exact test, and Mann–Whitney test were used to compare different groups. Missing data are listed in tables.

Cohens kappa (*k*) was calculated in order to test the agreement between MST, MNA-SF and the GLIM criteria. Cohens kappa values > 0.9 were considered almost perfect agreement, 0.81–0.90 strong, 0.61–0.80 moderate, 0.41–0.60 weak, < 0.4 minimal agreement [23].

## Results

**Fig. 1:**
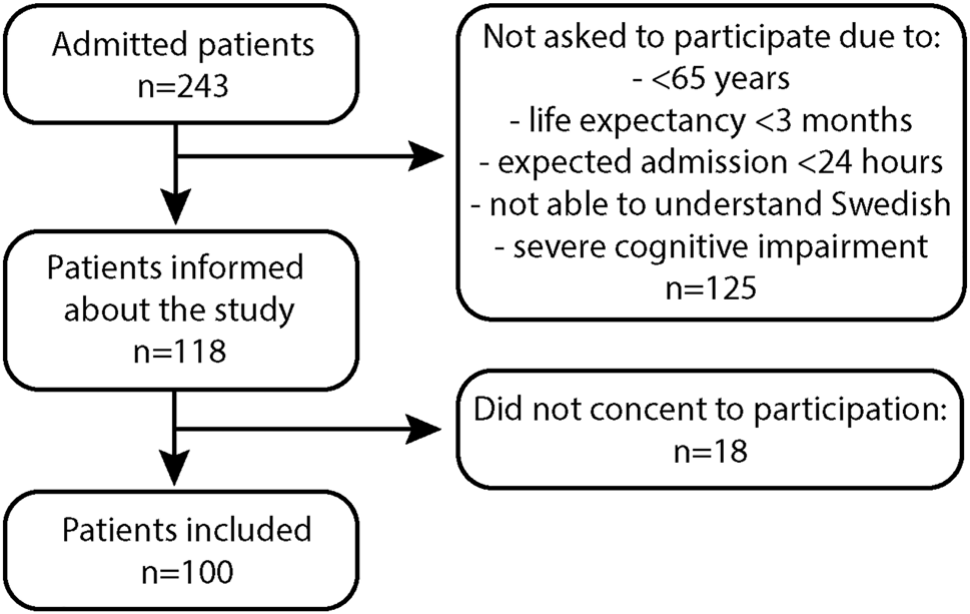
Flow diagram for inclusion

In this cross-sectional study a total of 100 patients (Fig. 1) with a mean age of 82 were included, of which 58% were female (Table 1). A total of 118 patients were asked to participate, and 100 consented to participation. Baseline characteristics for the total population (*n* = 100) and the two sub-samples are presented in Table 1.

**Table 1:** Baseline characteristics of all patients and according to localization

	Total	Sample A	Sample B	*p* value
	(*n* = 100)	(*n* = 50)	(*n* = 50)	
Sex, *n* (%)				
Women	65 (65)	33 (66)	32 (64)	1
Age (years), median (min–max)	83 (65–96)	84 (65–96)	83 (66–94)	0.38
BMI kg/m^2^, median (min–max)	24 (14–40)	24 (15–40)	29 (14–40)	0.37
MNA-SF				
Point score, median (min–max)	9 (3–12)	8 (3–12)	9 (4–12)	0.054
At risk of malnutrition, score ≤ 11 (%)	82 (87)	41 (89)	41 (85)	1
Malnutrition (score ≤ 7) (%)	30 (32)	21 (46)	9 (19)	0.008
Not at risk of malnutrition (score ≥ 12) (%)	12 (13)	5 (11)	7 (15)	0.76
Missing	6	4	2	
MST				
Point score, median (min–max)	1 (0–5)	1 (0–5)	2 (0–4)	0.15
At risk of malnutrition, score ≥ 2	47 (48)	20 (43)	27 (54)	0.31
Not at risk of malnutrition, score ≤ 1	50 (52)	27 (57)	23 (46)	0.55
Missing	3	3	0	
CRP mg/L, median (min–max)	25 (1–316)	42 (1–223)	17 (4–316)	0.038
Missing	2	0	2	
Energy intake (registered), median (min–max)	1462 (695–2313)	1408 (695–2313)	1550 (852–2216)	0.07
Missing	3	0	3	
Energy need, median (min–max)	1922 (1057–2862)	1928 (1057–2460)	1922 (1086–2862)	0.29
Main diagnosis (ICD-10 category), *n* (%)				
Neoplasms (C00-D48)	8 (8)	5 (10)	3 (6)	0.72
Circulatory system (I00–I99)	15 (15)	8 (16)	7 (14)	1
Respiratory system (J00–J99)	16 (16)	8 (16)	8 (16)	1
Musculoskeletal system and connective tissue (M00–M99)	16 (16)	3 (6)	13 (26)	0.012
Genitourinary system (N00–N99)	12 (12)	10 (20)	2 (4)	0.028
Injury, poisoning and certain other consequences of external causes (S00–T98)	8 (8)	2 (4)	6 (12)	0.27
Other main diagnoses^a^	22 (22)	12 (24)	11 (22)	1
Comorbidities, median (range)	5 (1–20)	8 (2–22)	4 (1–10)	< 0.001

^a^Other main diagnoses include ICD-10 categories: A00-B89, B99 Certain infectious and parasitic diseases, D50-D89 Diseases of the blood and blood-forming organs and certain disorders involving the immune mechanism, E00-E90 Endocrine, nutritional and metabolic diseases, F00-F99 other mental and behavioral disorders, G00-G99 epilepsy and other diseases of the nervous system, H60-H95 Diseases of the ear and mastoid process, K00-K93 Diseases of the digestive system, L00-L99 Diseases of the skin and subcutaneous tissue, R00-R99 Symptoms, signs and abnormal clinical and laboratory findings, not elsewhere classified, Z00-Z99 Factors influencing health status and contact with health services

Due to ethical and privacy considerations, no information was stored for the patients who were not asked to participate.

The two wards had a total of 243 patients admitted during the recruitment period meaning that 41% of the admitted patients were included in the study. There were no statistical differences in sex distribution, risk of malnutrition, or ratio of patients over the age of 80 years between the total admitted population and our sample.

The patients were screened for risk of malnutrition using both the MNA-SF and MST (Table 1). According to MNA-SF 82 patients were at risk of malnutrition, whereas using the MST 47 patients were at risk of malnutrition (Table 1).

When screening with MNA-SF more patients were detected as malnourished according to GLIM compared to when using the screening tool MST (Table 2). Excluding the screening tool before the use of the GLIM criteria, 58% of the patients were identified as malnourished (Table 2).

**Table 2:** Assessment criteria for malnutrition diagnosis according to GLIM (*n* = 100)

GLIM criterion		Patients fulfilling the criterion *n* (%)			*p* value MNA-SF vs MST
		GLIM no screening *n* = 58^g^	AFTER MNA-SF *n* = 51^g^	AFTER MST *n* = 35^g^	
Phenotypic	Low BMI^a^	28 (48)	25 (49)	15 (43)	0.66
	Unintentional weight loss, all	44 (76)	39 (76)	32 (91)	0.08
	Reduced muscle mass	35 (61)	30 (60)	19 (54)	0.66
	Low calf circumference, *n* (%)^b^	33 (73)	28 (72)	18 (72)	1
	Low mid arm circumference, *n* (%)^c^	2 (17)	2 (18)	1 (10)	1
Etiologic	Disease burden/inflammatory condition				
	Inflammation (CRP over 10 mg/L)	40 (70)	36 (72)	26 (76)	0.81
	WHO ICD-10 diagnosis^d^	50 (86)	43 (84)	31 (89)	0.75
	Reduced food intake or assimilation				
	Food intake, *n* < 50% (%)	2 (3)	2 (4)	1 (3)	1
	Symptoms of assimilation	24 (41)	19 (37)	12 (34)	0.88
	Nausea/vomiting, *n* (%)	7 (12)	6 (12)	5 (14)	0.75
	Diarrhea, *n* (%)	4 (7)	2 (4)	1 (3)	1
	Constipation, *n* (%)	8 (14)	8 (16)	6 (17)	1
	Dysphagia, *n* (%)	9 (16)	8 (16)	3 (9)	0.51
Mal-nutrition	Total malnutrition	58 (58)	51 (51)	35 (35)	0.032
	Moderate malnutrition, *n* (%)	29 (50)	25 (49)	13 (37)	0.37
	Severe malnutrition, *n* (%)	29 (50)	26 (51)	22 (63)	0.62
Physical function					
	Low hand grip strength^e^, *n* (%)	41 (72)	35 (70)	24 (69)	1
	Low 30-s-chair stand test^f^, *n* (%)	16 (80)	15 (88)	10 (91)	1
	Low modified 30-s-chair stand test^g^, *n* (%)	33 (89)	27 (82)	23 (96)	0.22

^a^Low BMI: BMI < 22 kg/m^2^ > 70 years, BMI < 20 kg/m^2^ for < 70 years

^b^Women under 32 cm, men under 33 cm. Adjusted for obesity as described in the method section

^c^Under 21 cm

^d^Clinical diagnosis of acute and chronic inflammatory disease in electronic patient record, e.g., major infections, burns, trauma (e.g., hip fracture) or closed head injury and other acute disease-/injury-related conditions associated with mild to moderate inflammation

^e^Cut-off values for hand grip strength: < 16/27 for women and men

^f^Cut-off values for 30-s-chair stand: Women 65–69 years: < 11; 70–79 years: < 10; 80–84 years: < 9; 85–89 years: < 8; 90–94 years: < 4; Men 65–69 years: < 12; 70–74 years: < 12; 75–79 years: < 11; 80–84 years: < 10; 85–89 years: < ; 90–94 years: < 7

^g^Percentages calculated from the n measured

The level of agreement between the methods were tested as kappa values and in general was found low. The kappa value for MNA-SF 0–7 vs a diagnosis of malnutrition according to GLIM was 0.314. The kappa value for MNA-SF 0–7 vs a diagnosis of severe malnutrition was 0.481. The kappa value for MST at risk of malnutrition (score 2–5) vs a diagnosis of malnutrition according to GLIM was 0.323. The kappa value for MST at risk of malnutrition (score 2–5) vs a diagnosis of severe malnutrition according to GLIM was 0.363. Among the malnourished patients according to GLIM, 3 patients were not at risk of malnutrition according to MNA-SF and 35 were not at risk of malnutrition according to MST.

Malnutrition according to GLIM using HGS in addition to calf circumference and mid arm circumference increased the number of patients with malnutrition to 69. If the muscle mass criteria was excluded the frequency of malnutrition is reduced to 53%.

The malnourished patients more often had weight losses, had significantly lower BMI, MST score, MNA-SF score, CC, and had more frequently HGS below the cut-offs, as compared to non-malnourished patients (Table 3). No statistical differences were found between the malnourished and non-malnourished for sex, age, CRP, 30-s-chair stand test nor reported eating difficulties (Table 3).

**Table 3:** Characteristics of malnourished and not malnourished patients

	No. malnutrition GLIM, *n* = 42	Malnutrition GLIM no screening, *n* = 58	*p* value
Sex, *n*			0.58
Women, *n* (%)	29 (69)	36 (62)	
Men, *n* (%)	13 (31)	22 (38)	
Age (years), median (min–max)	84 (69–96)	83 (65–93)	0.49
BMI median (min–max)	25.9 (20.5–40)	22.4 (13.9–38.4)	< 0.001
MST			
Point score, median (min–max)	1 (0–4)	2 (0–5)	< 0.001
MNA-SF			
Point score, median (min–max)	10 (4–12)	8 (3–12)	< 0.001
MNA-SF 0–7, *n* (%)	5 (12)	25 (43)	< 0.001
MNA-SF 12–14, *n* (%)	8 (19)	3 (5)	0.049
CRP mg/L, median (min–max)	20 (1–316)	36 (1–223)	0.87
Weight loss within 6 m			
5–10%, *n* (%)	2 (5)	22 (38)	< 0.001
Over 11%, *n* (%)	1 (2)	15 (26)	0.002
Weight loss over 6 month-2 years			
11–20%, *n*	2 (5)	14 (24)	0.012
Over 20%, *n*	1 (2)	10 (17)	0.023
Calf circumference			
median (min–max)	35 (31–44)	32 (21–39.5)	< 0.001
Men, median (min–max)	35.5 (31–44)	33 (31–39)	
Women, median (min–max)	34.5 (32–38)	30.5 (21–39.5)	
Mid arm circumference			
(when no CC), median (min–max)	32 (19–42)	30 (18–37)	0.3
Physical function			
Hand grip strength, all median (min–max)	18.5 (6–36)	18 (2–34)	0.1
Below cut-off, *n* (%)^a^ (women and men)	16 (40)	41 (75)	0.001
30-s-chair stand test, median (min–max)	0 (0–17)	0 (1–10)	0.67
Below cut-off^b^ (women and men)	6 (55)	16 (80)	0.22
Modified 30-s-chair stand test, median (min–max)	3 (0–11)	3 (0–12)	0.67
Below cut-off^b^ (women and men)	24 (86)	33 (89)	0.72
Energy intake and assimilation			
Energy intake (registered), median (min–max)	1514 (851–2216)	1452 (695–2313)	0.29
Energy need, median (min–max)	1973 (1525–2862)	1851 (1057–3250)	0.003
Reported eating difficulties and assimilation			
Nausea/vomiting, *n* (%)	10 (24)	7 (12)	0.18
Diarrhea, *n* (%)	1 (2)	4 (7)	0.4
Constipation, *n* (%)	5 (12)	8 (14)	1
Dysfagia, n (%)	9 (21)	9 (16)	0.6
WHO ICD-10 diagnosis, disease burden or inflammation, *n* (%)^c^	26 (62)	50 (86)	0.008

^a^Cut-off values for hand grip strength: < 16/27 for women and men

^b^Cut-off values for 30-s-chair stand: Women 65–69 years: < 11; 70–79 years: < 10; 80–84 years: < 9; 85–89 years: < 8; 90–94 years: < 4; Men 65–69 years: < 12; 70–74 years: < 12; 75–79 years: < 11; 80–84 years: < 10; 85–89 years: < ; 90–94 years: < 7

^C^Clinical diagnosis of acute and chronic inflammatory disease in electronic patient record, e.g., major infections, burns, trauma (e.g., hip fracture) or closed head injury and other acute disease-/injury-related conditions associated with mild to moderate inflammation

The feasibility of GLIM was tested as the completeness of the datasets, that is to which extent could the variables be assessed in all patients in our population. The variables BMI, unintentional weight loss, CRP, energy intake, assimilation and HGS were all feasible in our clinical setting (Table 4). The variable of reduced muscle mass measured by CC had a feasibility of 70% and was not suitable in the population due to a high frequency of edema, and for most of these patients MAC was used as an alternative proxy for muscle mass. For the 30-s-chair stand, 25% could not perform at least one even with the support of their arms, and 31% could not perform at least one without arm support (Table 4). No adverse events occurred during the collection of data (No fall, nausea, pain, was reported).

**Table 4:** Feasibility

Missing values		Total population* n* = 100 (*n*)	Feasibility (%)
GLIM criterion* Phenotypic*	Missing BMI	0	100
	Unintentional weight loss		
	Missing in EPR for within 6 months	4	96
	Missing in EPR for 6 months to 2 years	7	93
	Reduced muscle mass		
	Not able to perform (calf circumference)	30	70
	Not able to perform (either CC nor MAC)	5	17 (% of 30 missing CC)
GLIM criterion etiologic	Disease burden/inflammatory condition		
	Missing CRP	2	98
	Reduced food intake or assimilation		
	Missing food intake in EPR	3	97
Physical function	Hand grip strength	3	97
	30-s-chair stand		
	Not able to perform at least one without arm support	29	69
	Not able to perform at least one with arm support	25	75

## Discussion

### Summary of results

In our population of acute geriatric patients, 58% were malnourished according to GLIM when no prior screening for malnutrition was applied. With screening prior to GLIM, a higher rate of malnutrition was found after MNA-SF as compared to after MST, and thus the prevalence of malnutrition according to GLIM appears to be depending on the screening tool used. The phenotypic criterion of reduced muscle mass measured by CC had a low feasibility, while the rest of the GLIM criteria appeared feasible in clinical practice. Using handgrip strength as a proxy for reduced muscle mass even more of the patients were diagnosed with malnutrition.

### Prevalence of malnutrition

In another Swedish study in a geriatric clinic, Sobestiansky et al*.* found a very similar rate of malnutrition of 60% also by the GLIM using calf circumference and screening with MNA-SF [22]. Review of studies from 2012 to 2022 showed GLIM malnutrition rates between 11% (community dwelling older adults, no screening prior to GLIM [24] to 95% (geriatric rehabilitation, no screening prior to GLIM [25] in populations over 60 years of age, with the majority reporting malnutrition rates between 20 and 39% (19 of 38 studies) and only eight studies reporting malnutrition rates ≥ 50% [3]. Thus, the prevalence in our population is in line with other studies with similar populations, and in the high end of what is reported elsewhere, possibly since our hospitalized population is among the most vulnerable of the older adults.

### Screening as the first step in the GLIM process

There was a significant difference in malnutrition rates depending on whether MNA-SF or MST was performed prior to GLIM diagnostics. Other studies have also observed large variations in the rate of malnutrition according to the screening tool used [26–28]. To further complicate the screening step, the term “risk of malnutrition” remains to be clearly defined [29]. Furthermore, the agreement between both screening methods and malnutrition according to GLIM were weak to minimal. Clinicians and researchers should be aware that both the use of a screening tool or not, and the choice of screening tool can affect the rate of malnutrition within the GLIM process. For coming up-dates of the GLIM format for diagnosing malnutrition, the screening step will need certain considerations, in order to harmonize the process and to facilitate comparison. Our results indicate that in certain risk populations, like hospitalized geriatric patients, the screening step might be excluded, since almost all patients actually are at risk.

### Use of all the GLIM criteria

There is a fast growing pool of literature using the GLIM criteria for diagnosing malnutrition. However, the use of only selected GLIM criteria and different cut-off values, as well as multiple adaptations due to the datasets made in retrospective studies, complicate the comparison of studies. In the scoping review by Jobim Milanez et al*.* only 52% of the studies applied all five GLIM criteria when diagnosing malnutrition in hospital settings [30]. Current recommendations state that all five criteria shall be investigated when diagnosing malnutrition using GLIM [3] in the hospital setting. The phenotypic criterion most often not included is reduced muscle mass, while the etiological criterion most often left out of the evaluation is energy intake [30].

### The phenotypic criterion of reduced muscle mass

Even though the recent GLIM initiated guidance paper suggests CC and MAC for the estimations of muscle mass, in our study population CC was not a feasible measure due to the high frequency of edema [7]. Still, many of the patients fulfill several of the phenotypic criteria and are diagnosed with malnutrition irrespectively of the muscle mass criterion. In the hospital setting, 72% of the studies included the criterion of reduced muscle mass in diagnosing malnutrition according to GLIM, and the majority of these studies used anthropometric measures for muscle mass [30]. In our study, CC < 32/33 cm was used, while Sobestiansky et al*.* represented results with high specificity and high sensitivity between muscle mass CC < 31 cm and DEXA in the GLIM malnutrition diagnosis [22]. Furthermore, the MAC was used as a proxy in our study as suggested [7] but the selected cut off 21 cm [14] is very low, does not differ between men and women and has no adaptation for overweight and obesity.

Edema is highly frequent in older patients and can develop from malnutrition, renal-, hepatic- and cardiac diseases, and side effects of medication. To the best of our knowledge, few studies have investigated how edema influences CC and its use as a proxy for muscle mass. Ishida et al. found that older adults with lower extremity edema had CC increased by 1.6 cm in male and 2.0 cm in females compared to matched controls with no edema [31]. It should be kept in mind that all the phenotypic criteria of GLIM may be affected by edema, and thus, guidance should be established in the investigation of all phenotypic criteria in patients with edema.

There is an unmet need to further investigate the most optimal method to evaluate reduced muscle mass in older (hospitalized) patients with edema when direct measurement of muscle mass with DXA or BIA is not possible. Until such agreement is reached, the combination of CC and MAC, with HGS as a supportive measure when there is clinical concern or doubt, seems reasonable and feasible.

### Supportive measures for muscle mass

Few studies validating GLIM have applied direct body composition measures in older adults probably due to lack of equipment, resources, and retrospective designs. According to GLIM, is HGS only to be used as a supportive measure when diagnosing malnutrition, since HGS is not reflecting muscle mass but rather muscle function. We could not observe a significant difference in CC between GLIM positive (no prior screening) and patients with no malnutrition. Low HGS was frequent in our entire population, but significantly more frequent among malnourished patients. The high frequency of low HGS in both groups, but no difference in CC may indicate that HGS reflect the high prevalence of sarcopenia with reduced muscle function seen in aging [16]. This finding may also indicate that muscle function may not be affected by reduced muscle mass or related to malnutrition diagnosed with GLIM. Kaegi-Braun et al., conducted a secondary retrospectively analysis of the EFFORT trial and found that a modified version of GLIM using HGS for reduced muscle mass had a strong prognostic value for adverse clinical outcomes in adult hospitalized patients [6]. Contreras-Bolivar et al., concluded that GLIM criteria using HGS could predict 6-month mortality in inpatients with cancer [32]. The prevalence of low muscle strength measured with HGS in GLIM positive patients was approx. 19% in the EFFORT trial and 80% in the trial by Contreras-Bolivar et al*.* compared to 75% in our population. This indicates that low HGS is more frequent in patient populations where reduced muscle strength is expected due to high disease burden as seen in patients with cancer and geriatric patients.

Another measure of muscle function is the 30-s-chair stand, which was also lower in our malnourished patients, and in line with Aarden et al. who reported a very low 30-s-chair stand score for hospitalized patients [33].

### The etiological criteria

Disease burden was the only etiological criterion with a significant difference between the malnourished and the non-malnourished. In total, very few patients had an energy intake below the cut off despite a high frequency of nutrition impact symptoms. This might be because at these two clinics, all patients with risk of malnutrition have an individualized nutritional plan within 24 h of admission, and often at least two oral nutritional supplements (ONS) of 300 kcal a day. Our well documented energy intake is in contrast with the findings of Jobim Milanez et al. who concludes that there is unclear or unreported food intake in the majority of GLIM studies [30]. We use energy intake less than 50% compared to calculated energy needs and assimilation, both as reported in the electronic patient record. ESPEN recommend 30 kcal per kg body weight and day [34] whereas Alleparts et al. used indirect calorimetry and found that total energy expenditure were 24 kcal/kg and day for well-nourished and 28 kcal/kg for the undernourished [35]. It is therefore unlikely that other energy-calculations would affect the malnutrition rate to a greater extent in our study.

### Feasibility of the GLIM criteria in hospitalized geriatric patients

In general, the assessment of the majority of items included in GLIM were found to be feasible. The major exception was, not unexpected, the phenotypic criterion of reduced muscle mass, which is difficult to measure in clinical practice. The GLIM consortium has recommended various proxy tests, including calf circumference, as applicable in many clinical settings [7]. Still, CC that was used as the proxy measure for muscle mass was not feasible in this study. HGS is recommended by the same GLIM consortium to be measured in geriatric patients mainly for the purpose of diagnosing sarcopenia, but not for measuring malnutrition. HGS was feasible, but low in a very large part of the population, while the 30-s-chair stand was not feasible in our population of acute geriatric patients.

On the positive note, no adverse events were reported for any of the measurements of muscle mass or muscle function.

The feasibility part in our study did not include experiences or acceptability of GLIM from either patients, health care personnel or hospital managers about the measurements and the GLIM diagnosis. Future implementation research should also explore how resources, time, and commitment influence the use of the GLIM criteria.

### Strengths and limitations

The strengths in this study were that 100 patients were included in a prospective study, and only a few of the eligible patients declined to participate. We succeeded in recruiting patients, according to the power calculation. The recruited patients at the two wards were very similar, indicating that our findings might be generalized to other settings with geriatric patients.

All GLIM criteria were evaluated in every patient. Another strength is that the patient’s current energy intake was recorded for three days, and energy needs were calculated and adjusted. There were only a few missing data, almost all datasets were complete. However, our study was limited to the use of the equipment available at the wards. The precision of muscle mass estimations might have been higher were DXA, BIA or CT scans were available. A limitation in the study is that patients that could not participate due to language barriers or patients with severe cognitive impairment were not included in the study.

It can be considered both a strength and a limitation that our study was performed within daily clinical practice. A limitation since the variables are gathered by the daily ward staff, which may potentially introduce differences due to clinical evaluations. Our setting can also be considered a strength, since our study represents a very realistic view of what is feasible and manageable within daily geriatric practice and is likely to be comparable also to other settings with multimorbid older adults.

## Conclusion

In acute geriatric patients, the prevalence of malnutrition according to GLIM varied (35%, 51% and 58%) depending on the screening tool used. The GLIM criteria appear feasible in geriatric settings, besides edema related limitations for measure of the phenotypic criteria, as for calf circumference in this study. Future studies on the use of GLIM in geriatric settings should include longer term follow-up to look at the relationship between GLIM-defined malnutrition, nutritional treatment, and clinical outcomes.

## Data Availability

Data are subject to secrecy in accordance with the Swedish Public Access to Information and Secrecy Act, and thus cannot be made freely available, but can be made available to researchers upon request (requires application, approval and review of secrecy). Requests for data should be made to Anne-Marie Boström, e-mail: annemarie.bostrom@ki.se.
